# WHAT IS THE EFFECT OF REDUCING OBESITY ON LATER-LIFE DEPENDENCY? FINDINGS FROM THE PACSIM MODEL

**DOI:** 10.1093/geroni/igz038.192

**Published:** 2019-11-08

**Authors:** Andrew Kingston, Carol Jagger

**Affiliations:** 1 Newcastle University, Newcastle upon Tyne, United Kingdom; 2 Newcastle University, Newcastle upon Tyne, England, United Kingdom

## Abstract

Understanding the extent to which reducing obesity will help maintain independence in later life is of key importance in the goal to achieve healthy ageing. To investigate this we use a unique dynamic microsimulation model, the Population Ageing and Care Simulation (PACSim) model, formed from three Longitudinal studies: Understanding Society, the English Longitudinal Study of Ageing, the Cognitive Function and Ageing Study II; with the base population of 303,589 individuals aged 35 years and over (a 1% random sample of the England population in 2014). PACSim simulates the characteristics (sociodemographic factors, health behaviours including overweight and obesity, chronic diseases and geriatric conditions) of individuals between 2014 and 2040, with transition probabilities for characteristics estimated by modelling state changes from baseline to two-year follow-up in the combined studies. We estimate the effect of different strategies for obesity reduction on years spent independent from age 55 between 2015 and 2030.

